# Body mass index modulates the association between CDKAL1 rs10946398 variant and type 2 diabetes among Taiwanese women

**DOI:** 10.1038/s41598-018-31415-4

**Published:** 2018-09-05

**Authors:** Oswald Ndi Nfor, Ming-Fang Wu, Chun-Te Lee, Lee Wang, Wen-Hsiu Liu, Disline Manli Tantoh, Shu-Yi Hsu, Kuan-Jung Lee, Chien-Chang Ho, Tonmoy Debnath, Chia-Chi Lung, Yung-Po Liaw

**Affiliations:** 1 Department of Public Health and Institute of Public Health, Chung Shan Medical University, Taichung City, Taiwan; 2 School of Medicine, Chung Shan Medical University, Taichung City, Taiwan; 3 Divisions of Medical Oncology and Pulmonary Medicine, Chung Shan Medical University Hospital, Taichung City, Taiwan; 4 Department of Psychiatry, Chung Shan Medical University Hospital, Taichung City, Taiwan; 5 Department of Physical Education, Fu-Jen Catholic University, New Taipei City, Taiwan; 6 Department of Family and Community Medicine, Chung Shan Medical University Hospital, Taichung City, Taiwan

## Abstract

*CDKAL1* rs10946398 is a type 2 diabetes (T2D)-associated variant. It is a new body mass index (BMI)-associated variant in Asian populations. We investigated the association between rs10946398 and T2D among 9908 participants aged 30–70 years based on BMI: normal weight; 18.5 ≤ BMI < 24 kg/m^2^, overweight; 24 ≤ BMI < 27 kg/m^2^, and obesity; BMI ≥27 kg/m^2^. The CC genotype conferred a higher risk of T2D than the CA genotype. The odds ratios (ORs) were 1.83; 95% confidence interval (CI) 1.49–2.26 and 1.20; 95% CI 1.02–1.40, respectively. The C allele was the significant risk allele compared with A allele (OR = 1.32; 95% CI 1.19–1.47). For normal, overweight and obese participants with CC genotype, the ORs were respectively 1.69; 95% CI 1.02–2.81, 2.34; 95% CI 1.50–3.66, and 1.58; 95% CI 1.02–2.45 among men and 1.22; 95% CI 0.67–2.22, 2.42; 95% CI 1.30–4.52, and 2.3; 95% CI 1.19–4.50 among women. The C allele ORs were higher in obese and overweight women. In conclusion, the rs10946398 CC/CA genotypes, as well as the C allele increased the risk of T2D. The ORs were higher in women who were overweight and obese than in those with normal weight. Nonetheless, significant results were prominent only among those with CC genotype and C allele.

## Introduction

Type 2 diabetes is a complex metabolic disorder characterized by interaction between peripheral insulin resistance, hepatic glucose production, and beta-cell dysfunction. It remains an increasingly challenging public health issue. Globally, the prevalence of the disease is approximately 8.5% and nearly 592 million people are estimated to be infected by 2035^1,2^. In Taiwan, the prevalence is reported to have increased from 5.8% in 2000 to 12.4% in 2014^3^. High blood glucose is one of the significant risk factors for deaths among Taiwanese adults^4^.

Traditional risk factors have not fully accounted for the higher prevalence of diabetes^5^. Modifiable factors such as physical activity, maintenance of normal BMI, and dietary intake are relevant to studies investigating type 2 diabetes. About 36% of individuals with type 2 diabetes were found to be obese^6^. Besides the traditional risk factors, genetic determinants play a vital role in the development of type 2 diabetes. Some of the loci that have been consistently associated with the disease risk include *CDKAL1, HHEX, CDKN2A/2B*, *IGF2BP2*, *CDC123-CAMKID, TSPAN8-LGR5, THADA, SLC30A8, ADAMTS9, FTO, PCSK9*, and *NOTCH2*, with odds ratios ranging from 1.09–1.79^7–10^. New independent risk variants have also been reported^11^. Most of the studies investigating the association between genes and T2D have been carried out in Europe^12^. However, only a few genetic markers for type 2 diabetes have been reported in Asia^11^.

Cyclin-dependent kinase 5 regulatory subunit-associated protein 1-like 1 (*CDKAL1*) is among the genes consistently investigated for diabetes susceptibility among Europeans. One of its variants (rs10946398) has been significantly associated with T2D^9,12–15^, gestational diabetes^16^ and diabetic retinopathy^17^ in different subgroups. The C allele of rs10946398 is reported to be a T2D risk allele^18^. A meta-analysis of cohort studies has reported significant associations between *CDKAL1* variant and T2D in general populations^19^. However, further analyses showed significant associations only in Asian but not African subgroups. This is an indication that results from individual studies are not consistent with one another.

Obesity (defined by BMI) is strongly associated with the risk of type 2 diabetes^20^. There are sex differences in the pathogenesis of this disease. For example, men are said to develop it at a relatively lower BMI than women^21^. Genetic components have different effects in men and women. However, most of the previous associations have focused only on one sex. SNP rs10946398 is one of the variants that have shown the strongest associations with diabetes particularly in European and South East Asian populations. Besides, it is a new BMI-associated locus specifically among Asian populations^22^. However, it has received less attention than other SNPs^22^. To date, only one study has investigated the effect of this SNP on T2D in Taiwan^12^. Using Taiwan biobank resources, we assessed whether there are sex-related differences in the association between rs10946398 SNP and T2D based on BMI.

## Results

Table 1 presents the basic characteristics of study participants and odds ratios of type 2 diabetes. Among 974 participants identified with type 2 diabetes, 619 were men and 355 were women. The mean age was 48.60 years (SD = 10.99) for men and 48.64 years (SD = 10.63) for women. The risk of diabetes imparted by the CC genotype (OR = 1.83; 95% CI, 1.49–2.26) was higher than that of the CA genotype (OR = 1.20; 95 CI, 1.02–1.40). In addition, the C allele’s effect was more significant compared with the A allele (OR = 1.32; 95% CI 1.19–1.47). The odds ratio for T2D was higher among men compared to women (OR = 1.20; 95% CI, 1:00–1.44). Table 2 presents the baseline characteristics of study participants stratified by BMI. The proportion of individuals with T2D were significantly different (p < 0.0010). Obese individuals had the highest rate of diabetes (19.64%) compared with the overweight (10.01%) and normal weight (5.42%) individuals.

**Table 1:** Basic characteristics of study participants and odds ratios of type 2 diabetes.

	Basic characteristics of participants			Risk of type 2 diabetes	
	Diabetes	No diabetes	P-value	OR	(95% CI)
rs10946398 (N, %)			<0.0001		
AA	353 (36.24)	3707 (41.49)		ref.	—
CA	441 (45.28)	4061 (45.46)		1.20	1.02–1.40
CC	180 (18.48)	1166 (13.05)		1.83	1.49–2.26
Allele			<0.0001		
A	1147 (58.88)	11475 (64.22)		ref	
C	801 (41.12)	6393 (35.78)		1.32	1.19–1.47
Sex (N, %)			<0.0001		
Men	619 (63.55)	4483 (50.18)		ref.	—
Women	355 (36.45)	4451 (49.82)		1.20	1.00–1.44
Age (years)	55.56 ± 9.19	47.60 ± 10.80	<0.0001	1.07	1.06–1.08
BMI			<0.0001		
18.5 ≤ BMI < 24	260 (26.69)	4534 (50.75)		ref.	—
24 ≤ BMI < 27	302 (31.01)	2714 (30.38)		1.30	1.08–1.57
≥27	412 (42.3)	1686 (18.87)		3.16	2.60–3.83
T-CHO (mg/dl)	189 (49)	191 (46)	0.0237	0.98	0.98–0.99
TG (mg/dl)	132 (97)	93 (69)	<0.0001	1.01	1.00–1.01
HDL-C (mg/dl)	45 (14)	52 (18)	<0.0001	0.98	0.97–1.00
LDL-C (mg/dl)	118 (45)	120 (41)	0.0092	1.01	1.00–1.02
Uric acid (mg/dl)	5.96 (1.48)	121.90 (31.45)	<0.0001	0.86	0.82–0.92
SBP (mmHg)	126.60 (17.41)	115.10 (16.55)	<0.0001	1.02	1.02–1.03
DBP (mmHg)	75.92 (11.02)	72.06 (11.02)	<0.0001	0.98	0.98–0.99
Drinking (N, %)			0.0340		
No	875 (89.84)	8203 (91.82)		ref.	—
Yes	99 (10.16)	731 (8.18)		0.99	0.76–1.27
Smoking (N, %)			0.004		
No	827 (84.91)	8203 (91.82)		ref.	—
Yes	147 (15.09)	1063 (11.90)		1.10	0.88–1.37
Physical activity (N, %)			<0.0001		
No	489 (50.21)	8203 (91.82)		ref.	—
Yes	485 (49.79)	731 (8.18)		1.04	0.89–1.22

Normally distributed variables are presented as mean (SD) and compared by t-test. Non-normally distributed variables are presented as median (interquartile range [IQR]) and compared by Wilcoxon rank-sum test. Other variables are presented as numbers (%). SD: Standard deviation; BMI: Body mass index (measured in Kg/m^2^); T-CHO: Total cholesterol; HDL-C: High-density lipoprotein cholesterol; LDL-C: Low-density lipoprotein cholesterol; SBP: Systolic blood pressure; DBP: Diastolic blood pressure; OR: Odds ratio; CI: Confidence interval.

**Table 2:** Characteristics of study participants according to body mass index.

	BMI classes			
	18.5 ≤ BMI < 24 n = 4794	24 ≤ BMI < 27 n = 3016	BMI ≥ 27 n = 2098	P-value
Diabetes mellitus (N, %)				<0.0010
No	4534 (94.58)	2714 (89.99)	1686 (80.36)	
Yes	260 (5.42)	302 (10.01)	412 (19.64)	
rs10946398 genotype (N, %)				0.1560
AA	1958 (40.84)	1213 (40.22)	889 (42.37)	
CA	2152 (44.89)	1399 (46.39)	951 (45.33)	
CC	684 (14.27)	404 (13.40)	258 (12.30)	
Allele				0.1242
A	6068 (63.29)	3825 (63.41)	2729 (65.04)	
C	3520 (36.71)	2207 (36.59)	1467 (34.96)	
Gender				<0.0010
Men (N, %)	1872 (39.05)	1896 (62.86)	1334 (63.58)	
Women (N, %)	2922 (60.95)	1120 (37.14)	764 (36.42)	
Age (years)	47.57 (10.96)	50.12 (10.77)	47.72 (10.73)	<0.0010
T-CHO (mg/dl)	188 (44)	194 (46.5)	192 (45)	<0.0001
TG (mg/dl)	79 (53)	108 (76.5)	130 (93)	<0.0001
HDL-C (mg/dl)	56 (17)	49 (14)	45 (13)	<0.0001
LDL-C (mg/dl)	115 (40)	124 (41)	125 (42)	<0.0001
Uric acid (mg/dl)	5.18 (1.32)	6.02 (1.42)	6.43 (1.52)	<0.0010
SBP (mmHg)	111.33 (16.23)	119.31 (16.22)	123.07 (16.35)	<0.0010
DBP (mmHg)	68.88 (10.28)	74.35 (10.48)	77.81 (10.86)	<0.0010
Drinking (N, %)				
No	4498 (93.83)	2713 (89.95)	1867 (88.99)	
Yes	296 (6.17)	303 (10.05)	231 (1 1.01)	
Smoking (N, %)				<0.0010
No	4340(90.53)	2615(86.7)	1743 (83.08)	
Yes	454 (9.47)	401 (13.3)	355(16.92)	
Physical activity (N, %)				<0.0010
No	2819 (58.8)	1727 (57.26)	1347 (64.20)	
Yes	1975 (41.2)	1289 (42.74)	751 (35.80)	

Normally distributed variables are presented as mean (SD) and compared by ANOVA. Non-normally distributed variables are presented as median (interquartile range [IQR]) and compared by Kruskal-Wallis test. Other variables are presented as numbers (%). SD: Standard deviation; BMI: Body mass index (measured in Kg/m^2^); T-CHO: Total cholesterol; TG: Triglyceride; HDL-C: High-density lipoprotein cholesterol; LDL-C: Low-density lipoprotein cholesterol; SBP: Systolic blood pressure; DBP: Diastolic blood pressure; OR: Odds ratio; CI: Confidence interval.

Table 3 shows the association of rs10946398 with type 2 diabetes across different categories of BMI. The risks imparted by the CC genotype were as follows: normal weight (OR = 1.47; 95% CI, 1.01–2.15), overweight (OR = 2.33; 95% CI, 1.62–3.33), and obesity (OR = 1.80; 95% CI, 1.25–2.57), respectively. For the CA genotype, a significant association was found only among obese individuals (OR, 1.31; 95% CI, 1.02–1.69). However, only a borderline association was found among overweight individuals (OR, 1.33, p = 0.0540). The C allele odds ratios (compared with the A allele) were 1.17 (0.97–1.41), 1.50 (1.25–1.79), and 1.33 (1.13–1.58) for normal weight, overweight and obese individuals. In the dominant model, the interaction between BMI and rs10946398 on T2D risk was significant among women (P for interaction = 0.0318), but not men (P for interaction = 0.969).

**Table 3:** Association of rs10946398 with type 2 diabetes stratified by BMI.

	BMI classes								
	18.5 ≤ BMI < 24			24 ≤ BMI < 27			BMI ≥ 27		
	OR	95% CI	P-value	OR	95% CI	P-value	OR	95% CI	P-value
Genotype									
AA	ref.	—		ref.	—		ref.	—	
CA	0.99	0.74–1.33	0.9530	1.33	1.00–1.76	0.0540	1.31	1.02–1.69	0.0340
CC	1.47	1.01–2.15	0.0450	2.33	1.62–3.33	<0.0001	1.80	1.25–2.57	0.0010
Allele									
A	ref.			ref.			ref.		
C	1.17	0.97–1.41	0.1058	1.50	1.25–1.79	<0.0001	1.33	1.13–1.58	0.009
Gender									
Women	ref.	—		ref.	—		ref.	—	
Men	2.06	1.48–2.86	<0.0001	0.80	0.58–1.11	0.1770	1.08	0.81–1.44	0.6250
Age	1.09	1.07–1.11	<0.0001	1.07	1.06–1.09	<0.0001	1.06	1.04–1.07	<0.0001
T-CHO	0.99	0.97–1.00	0.0500	0.99	0.97–1.00	0.0180	0.98	0.97–0.99	0.0010
TG	1.01	1.00–1.01	<0.0001	1.00	1.00–1.01	0.0020	1.01	1.00–1.01	<0.0001
HDL-C	0.99	0.97–1.01	0.2060	0.98	0.96–0.99	0.0080	0.99	0.98–1.01	0.4690
LDL-C	1.01	1.00–1.03	0.0900	1.01	0.99–1.02	0.3850	1.02	1.00–1.03	0.0130
Uric acid	0.86	0.76–0.96	0.0090	0.90	0.81–1.00	0.0490	0.83	0.76–0.90	<0.0001
SBP	1.02	1.01–1.04	<0.0001	1.02	1.01–1.03	<0.0001	1.02	1.01–1.03	<0.0001
DBP	0.98	0.96–1.00	0.0120	0.98	0.97–1.00	0.0450	0.99	0.98–1.01	0.2950
Drinking									
No	ref.	—		ref.	—		ref.	—	
Yes	0.71	0.39–1.31	0.2760	1.29	0.85–1.98	0.2360	1.02	0.66–1.43	0.8870
Smoking									
No	ref.	—		ref.	—		ref.	—	
Yes	1.07	0.68–1.69	0.7770	1.22	0.83–1.80	0.3210	1.01	0.72–1.41	0.9640
Physical activity									
No	ref.	—		ref.	—		ref.	—	
Yes	1.23	0.92–1.65	0.1600	0.91	0.69–1.19	0.4740	1.01	0.79–1.30	0.9290

BMI: body mass index (measured in Kg/m^2^); OR: odds ratio; CI: confidence interval.

Table 4 shows the association between rs10946398 and type 2 diabetes stratified by sex and BMI. There was no significant interaction between rs10946398 and sex. The risk of T2D among men with CC genotype was as follows: OR = 1.69 (95% CI, 1.02–2.81) among normal weight, 2.34 (95% CI, 1.50–3.66) among overweight and 1.58 (95% CI, 1.02–2.45) among obese individuals. Association between the CA genotype and T2D was not significant regardless of BMI: The odds ratios were 1.16 (CI 0.78–1.72), 1.16 (CI 0.81–1.66), and 1.23 (CI 0.90–1.69) among the normal weight, overweight, and obese category, respectively. Normal weight, overweight and obese women with CC genotype had odds ratios of 1.22 (95% CI, 0.67–2.22), 2.42 (95% CI, 1.30–4.52), and 2.31 (95% CI, 1.19–4.50), respectively. Furthermore, normal weight, overweight, and obese women with the CA genotype had odds ratios of 0.77 (CI 0.49–1.21), 1.59 (CI 0.97–2.60), and 1.51 (CI 0.98–2.32), respectively. The risk of T2D conferred by the C allele was more prominent in overweight (OR = 1.56 [CI 1.15–2.12]) and obese (OR = 1.54 [CI 1.13–2.07]) women compared to those with normal weight (OR = 1.02 [0.75–1.37]). The C allele odds ratios were 1.28 (0.99–1.64), 1.47 (1.18–1.85), and 1.25 1.02–1.54) for normal weight, overweight, and obese men. For menopausal women, a significantly higher risk was found only in the normal weight category (OR, 2.61; 95% CI, 1.17–5.80). Broadly speaking, the risk of T2D conferred by the CC genotype was higher than that of the CA genotype regardless of sex. The CC vs CA odds ratio was 1.69 vs 1.16 for normal weight, 2.34 vs 1.16 for overweight and 1.58 vs 1.23 for the obese men. Likewise, the ratios were 1.12 vs 0.77 for normal weight, 2.42 vs 1.59 for overweight and 2.31 vs 1.51 for obese women, respectively. Smoking, physical activity, and drinking were not significantly associated with T2D.

**Table 4:** Multiple logistic regression analysis of type 2 diabetes in relation to rs10946398 among Taiwanese men and women.

	Men						Women					
	18.5 ≤ BMI < 24		24 ≤ BMI < 27		BMI ≥ 27		18.5 ≤ BMI < 24		24 ≤ BMI < 27		BMI ≥ 27	
	OR	95% CI	OR	95% CI	OR	95% CI	OR	95% CI	OR	95% CI	OR	95% CI
Genotype												
AA	ref.	—	ref.	—	ref.	—	ref.	—	ref.	—	ref.	—
CA	1.16	(0.78–1.72)	1.16	(0.81–1.66)	1.23	(0.90–1.69)	0.77	(0.49–1.21)	1.59	(0.97–2.60)	1.51	(0.98–2.32)
CC	1.69	(1.02–2.81)	2.34	(1.50–3.66)	1.58	(1.02–2.45)	1.22	(0.67–2.22)	2.42	(1.30–4.52)	2.31	(1.19–4.50)
Allele												
A	ref.		ref.		ref.		ref.		ref.		ref.	
C	1.28	(0.99–1.64)	1.47	(1.18–1.85)	1.25	(1.02–1.54)	1.02	(0.75–1.37)	1.56	(1.15–2.12)	1.54	(1.13–2.07)
Age	1.08	(1.06–1.11)	1.07	(1.05–1.09)	1.06	(1.04–1.07)	1.05	(1.01–1.09)	1.07	(1.03–1.12)	1.05	(1.01–1.09)
T-CHO	0.98	(0.96–1.00)	0.99	(0.97–1.00)	0.98	(0.97–1.00)	0.99	(0.97–1.01)	0.97	(0.94–0.99)	0.98	(0.96–0.99)
TG	1.01	(1.00–1.01)	1.00	(1.00–1.01)	1.01	(1.00–1.01)	1.01	(1.00–1.01)	1.01	(1.01–1.02)	1.01	(1.01–1.01)
HDL-C	1.00	(0.97–1.03)	0.97	(0.95–1.00)	0.99	(0.96–1.01)	0.98	(0.95–1.01)	1.00	(0.97–1.04)	1.01	(0.98–1.04)
LDL-C	1.02	(1.00–1.04)	1.00	(0.98–1.01)	1.01	(1.00–1.03)	1.01	(0.99–1.04)	1.03	(1.01–1.06)	1.02	(0.99–1.04)
Uric acid	0.69	(0.60–0.80)	0.84	(0.74–0.95)	0.74	(0.66–0.82)	1.26	(1.04–1.53)	1.03	(0.86–1.24)	1.07	(0.92–1.26)
SBP	1.03	(1.02–1.05)	1.02	(1.01–1.04)	1.03	(1.01–1.04)	1.01	(0.99–1.03)	1.02	(1.01–1.04)	1.01	(0.99–1.03)
DBP	0.96	(0.94–0.99)	0.97	(0.95–0.99)	0.99	(0.97–1.01)	0.99	(0.96–1.02)	1.00	(0.97–1.03)	1.01	(0.98–1.03)
Drinking												
No	ref.	—	ref.	—	ref.	—	ref.	—	ref.	—	ref.	—
Yes	0.62	(0.32–1.22)	1.38	(0.89–2.13)	1.05	(0.70–1.56)	1.43	(0.32–6.39)	0.94	(0.09–9.49)	0.37	(0.04–3.14)
Smoking												
No	ref.	—	ref.	—	ref.	—	ref.	—	ref.	—	ref.	—
Yes	1.28	(0.88–1.86)	1.09	(0.78–1.51)	0.99	(0.73–1.35)	0.48	(0.06–3.73)	1.50	(0.41–5.50)	1.37	(0.40–4.66)
Physical activity												
No	ref.	—	ref.	—	ref.	—	ref.	—	ref.	—	ref.	—
Yes	1.53	(1.02–2.28)	0.95	(0.67–1.33)	0.93	(0.68–1.26)	1.05	(0.67–1.64)	0.86	(0.55–1.36)	1.20	(0.79–1.82)
Menopause												
No	—	—	—	—	—	—	ref.	—	ref.	—	ref.	—
Yes	—	—	—	—	—	—	2.61	(1.17–5.8)	0.96	(0.46–2.02)	1.21	(0.60–2.44)

BMI, body mass index (measured in Kg/m^2^); OR, odds ratio; CI, confidence interval.

## Discussion

In this study, we found a significant association between *CDKAL1* rs10946398 and type 2 diabetes among Taiwanese individuals. The association was substantially stronger among CC compared to CA carriers, as well as in C allele compared to A allele carriers. Based on stratified analyses, the CC genotype was significantly associated with T2D in overweight and obese women, as well as in men regardless of their BMI. Several studies have provided evidence for the significant contribution of *CDKAL1* rs10946398 to T2D risk^13,14,16,22,23^. According to findings from a global meta-analysis, 8 studies have reported a trend of elevated ORs for the C risk allele, whereas only two studies have found no associations^14^.

In our initial analysis, the CA genotype was a risk factor for T2D. However, after stratification by BMI, a significant odds ratio was found only among obese individuals (OR, 1.31; 95% CI, 1.02–1.69). When stratified by sex, borderline associations were found among overweight (OR, 1.59, P = 0.064) and obese (OR, 1.51, P = 0.061) women. However, significant odds ratios were not observed in men.

Menopause has been associated with certain unfavorable changes in the body that serve as predisposing factors for type 2 diabetes^24,25^. Post-menopausal women are believed to be more susceptible to T2D due to their greater percentage of body fat and intra-abdominal visceral fat^26^. In the present study, an increased risk of T2D was found among normal weight menopausal women (OR, 2.61; 95% CI, 1.17–5.80).

Based on the criteria defined by the Department of Health, a BMI of 18.5–23.9 kg/m^2^ indicates normal weight, 24–26.9 kg/m^2^ indicates overweight while ≥27 kg/m^2^ indicates obesity^27^. The cut-point for T2D risk in different Asian populations varies from 22–25 kg/m^2^. Values above 26 kg/m^2^ are associated with a higher risk of T2D^28^. In a study conducted in Taiwan, Chang and colleagues reported that the rs10946398 C allele was associated with an increased risk of T2D. This association was attenuated in persons with a larger BMI^12^. Nonetheless, we found contrasting results. In their study, the sample size was comparatively small and stratifications did not include sex. In addition, there was no information about the total number of participants with a higher BMI (>26.9 kg/m^2^). In another study, *ENPP1* rs1044498 was significantly associated with T2D but not with obesity^29^. It is worth mentioning that T2D had no significant association with smoking, physical activity, and alcohol drinking.

The mechanism through which the *CDKAL1* gene influences T2D is not fully understood. However, it has been reported that it codes for the cyclin-dependent kinase 5 (*CDK5)* regulatory subunit-associated protein 1-like 1 which may affect the activity of the *CDK5* protein^15^. This may subsequently lead to degeneration of β cells and development of type 2 diabetes mellitus^14^.

In this Taiwan-based study, we have attempted to validate the association between *CDKAL1* rs10946398 and type 2 diabetes. Such findings are necessary considering that T2D is one of the significant risk factors associated with mortality in Taiwan. Furthermore, noting that *SNP* rs10946398 is a new BMI-associated locus specifically for individuals of Asian ancestry, large dedicated studies are necessary to confirm such associations^22^. Genetic variants associated with BMI may also have associations with metabolic traits. Stratification of samples helps to clarify the role of BMI in modulating pancreatic beta-cell function possibly influenced by the rs10946398 variant. So far, such stratifications have not been widely considered in Asian populations^13^. Our study is limited in that we used a nonrandom or purposive sampling technique; hence, results may not be fully reflective of the general population. Second, there is a possibility of a response bias considering that information were collected using questionnaires. Moreover, there was no information regarding diabetes regimens. Finally, multiple stratifications might have affected our results. From this aspect, there is a need for additional research.

In summary, our study provides the following conclusions: (1) there is a significant association between *CDKAL1* rs10946398 and type 2 diabetes among Taiwanese men and women. (2) The CC genotype is a risk factor for type 2 diabetes in women with BMI ≥ 24 kg/m^2^, as well as in men regardless of their BMI. (3) The CA genotype appears to be a risk factor for T2D mainly in obese individuals. In conclusion, we found that rs10946398 CC/CA genotypes and C allele increased the risk of T2D among Taiwanese adults. Overweight and obese women had higher odds ratios than normal women. However, the effect was significant only among those with CC genotype and C allele. These findings should serve as an incentive for larger dedicated studies.

## Methods

### Data source

Data were obtained from Taiwan Biobank, a database with genetic information of over 89169 individuals aged 30–70 years^30–32^. The project aims to recruit over 200,000 volunteers by 2027^32^. All methods were carried out in accordance with the relevant guidelines and regulations. Participants obtained written informed consents prior to data collection. The study protocol was approved by the Institutional Review Board of Chung Shan Medical University.

### Study participants

Overall, 10853 Taiwan Biobank participants aged 30–70 years were recruited. Excluded were individuals with missing information (n = 652) and those with BMI under 18 (n = 93). The final enrollment included 9908 participants with complete phenotypic and genotypic data. Taiwan Biobank has 29 recruitment centers. Participants were assessed in specific recruitment centers during the period 2012–2016. Among those selected, 355 women and 619 men had type 2 diabetes. Individuals were defined as having type 2 diabetes mellitus if they (1) had a fasting glucose level ≥126 mg/dl; (2) had a glycosylated hemoglobin A1c value of at least 6.5%); or (3) self-reported a history of diabetes based on the question, “have you ever been diagnosed with type 1 or type 2 diabetes by a doctor or health professional?” In general, 49.49% (n = 482) of patients with type 2 diabetes were diagnosed by physicians. Participants were grouped into BMI categories: normal weight (18.5 ≤ BMI < 24 kg/m^2^), overweight (24 ≤ BMI < 27 kg/m^2^), and obesity (BMI ≥ 27 kg/m^2^). The selected phenotypic characteristics included age, sex, body mass index, total cholesterol (T-CHO), triglycerides (TG), high-density lipoprotein (HDL-C), low-density lipoprotein (LDL-C), uric acid, systolic blood pressure (SBP), and diastolic blood pressure (DBP). Lifestyle factors included physical activity, smoking (never/former and current smoker), and alcohol consumption (150 c.c per week or on a regular basis for 6 months). Information on menopause was based on self-report. Women who reported a complete absence of menstrual period for 12 consecutive months without hysterectomy were categorized as naturally menopausal. These variables were selected based on their previous assessments in previous studies^33,34^.

### Genetic variant selection/Genotyping

We searched peer-reviewed literature databases (Pub Med, ScienceDirect, Google Scholar, SNPedia, and GWAS Catalog) to identify common *CDKAL 1* gene variants that have been associated with type 2 diabetes. Four of the SNPs (rs10946398, rs7754840, rs7756992, and rs9465871) were chosen for analysis. However, only rs10946398 was included in the final analysis because of its highly significant association. Furthermore, it is the only CDKAL1 SNP associated with BMI among Asian populations. In addition, it has been shown to have a more striking replication signal. SNP genotyping was performed at the National Center for Genome Medicine in Academia Sinica using the Axiom-Taiwan Biobank Array Plate (Affymetrix, Santa Clara, CA, USA)^31^. The Axiom™ Genome-Wide ASI Array Plate maximizes genomic coverage of common and rare alleles of East Asian genome while the Axiom™ Genome-Wide CHB Array plate maximizes genomic coverage of common alleles of the Han Chinese genome. Only participants with call rates greater than 90% were included in the study. SNPs were excluded if the minor allele frequency (MAF) was <0.05. Also excluded were SNPs whose genotypes deviated from the Hardy-Weinberg equilibrium (HWE).

### Statistical analysis

We used the PLINK 1.09 beta and SAS 9.3 software (SAS Institute, Cary, NC) for data management and statistical analyses. The distribution of variables were tested using Kolmogorov-Smirnov test and Shapiro-Wilk test. Data with normal distributions were analyzed by t-test and ANOVA while those not normally distributed were compared by Wilcoxon rank-sum test and Kruskal-Wallis test. Normally distributed variables were presented as mean and standard deviation (SD) while non-normally distributed variables (T-CHO, TG, HDL-C, and LDL-C) were presented as median and interquartile range (IQR). Genotypic associations of the SNP with T2D were determined using the Chi-square test. The risk allele/genotype-specific odds ratios and 95% confidence intervals were calculated using multivariate logistic regression models. Associations of genotypes with T2D were represented as co-dominant models. The interaction between BMI and rs10946398 on T2D risk was tested using a dominant model.

## Data Availability

The data that support the findings of this study are available from Taiwan Biobank Institutional Dataset. To gain access, interested individuals should contact “biobank@gate.sinica.edu.tw”.
